# Improved Self-Reported Comfort, Stability, and Limb Temperature Regulation With an Immediate Fit, Adjustable Transtibial Prosthesis

**DOI:** 10.1016/j.arrct.2020.100090

**Published:** 2020-11-02

**Authors:** Chloe McCloskey, Jessica Kenia, Frances Shofer, Jim Marschalek, Timothy R. Dillingham

**Affiliations:** a Department of Physical Medicine and Rehabilitation, University of Pennsylvania Perelman School of Medicine, Philadelphia, PA; b Department of Emergency Medicine, University of Pennsylvania Perelman School of Medicine, Philadelphia, PA; c Advanced Design Concepts, Pewaukee, WI

**Keywords:** Amputation, Prosthesis, Rehabilitation, PEQ, Prosthetic Evaluation Questionnaire

## Abstract

**Objective:**

The purpose of this investigation was to assess participants’ self-reported satisfaction with an adjustable, immediate fit transtibial prosthetic system as compared to their conventionally fabricated prosthetic device.

**Design:**

A prospective study involving a 2-week single-group pre-post intervention design.

**Setting:**

Physical medicine and rehabilitation clinic of a university hospital.

**Participants:**

Adults (N=27) with transtibial limb loss.

**Intervention:**

Participants were fit with the iFIT prosthetic system and instructed to wear it for a 2-week evaluation period.

**Main Outcome Measure:**

A modified Prosthetic Evaluation Questionnaire (PEQ) scale was completed on the participant’s conventional prosthetic during the initial visit and the iFIT system after 2 weeks.

**Results:**

Twenty-seven persons with lower limb loss were enrolled. Three were lost to follow-up leaving 24 participants with completed data. Three participants had recent amputations with no conventional device for comparison. The modified PEQ scores were significantly higher for the iFIT prosthetic in comparison to their conventional device (29.18±4.63 vs 23.82±6.38, P<.01). Participants were also found to perceive significantly better temperature control with the iFIT prosthetic system (4.19±0.68 vs 2.97±1.02, P<.001). Participants did not report any skin breakdown, prosthetic issues, or falls.

**Conclusion:**

This immediate fit, adjustable transtibial prosthesis demonstrated significantly better patient satisfaction and temperature perception compared to conventional devices. These results are consistent with previous findings and further support the efficacy of an immediate fit adjustable transtibial prosthetic system. Longer-term studies in the United States and internationally are underway to assess the durability and efficacy of this new prosthesis in different populations and settings.

Lower limb amputations are disabling conditions that most frequently result from diabetes, peripheral vascular disease, and trauma.^1^, ^2^, ^3^, ^4^ In 2005, it was estimated that 1.6 million people in the United States were living with lower limb loss, and this is projected to double to 3.6 million by the year 2050.^1^ Rates of amputations among dysvascular patients over the past 2 decades are 8 times greater than the rate of traumatic amputations.^4^ There have been care guideline and quality metrics developed to reduce dysvascular-related amputations through education, specialized clinics, medical homes, and treatment guidelines. These have had some effect; however, there are still large numbers of people incurring dysvascular lower limb loss each year.^3^^,^^5^^,^^6^

Mackenzie et al^7^ found the estimated lifetime health care cost for patients who have undergone lower extremity amputation at any level to be approximately $500,000.^7^ Within this 2007 study, the average cost for a transtibial socket was found to be $10,058, which is expected to be higher now when considering rates of inflation.^7^ Increasingly, private insurance companies are reducing prosthetic benefits or issuing an annual *insurance cap* on prosthetic services ranging from $500 to $3000,^3^ which severely limits a patient’s postamputation prosthetic options. In addition, in the developing world, it is estimated that 80% of those needing a lower limb prosthesis are unable to afford it, even if prosthetic services were available.^8^

Traditionally, lower limb prostheses are made through a fabrication process that involves casting a patient’s residual limb, and creating a positive mold of the limb that is then used to create test sockets out of thermomolded plastics. Finally, a hard socket made from laminated materials is created from the optimal positive mold.^9^ This process often takes weeks or months to complete. Patients frequently experience significant changes in limb volume and size once initiating gait training, requiring further adjustments to the inner liner, addition/removal of socks, and grinding out or making cutouts in the sockets. In many cases, the process of fabricating a new socket altogether must be undertaken to accommodate limb changes. Hard sockets lack the adjustability to provide comfort. One study of persons with traumatic amputation found that only 43% were satisfied with the comfort of their prosthesis.^10^

The iFITa prosthesis was developed as an economical socket that can be mass produced and fit immediately to the patient. The iFIT transtibial prosthesis can be fit in a single session using a few hand tools. The socket is injection molded with advanced polymer materials and can be readily fit and aligned to patients in one setting (fig 1). An array of transtibial socket sizes based on residual limb length and distal circumference are available to fit most residual limbs. The socket circumferences are adjustable using a locking buckle system. A more customized fit is addressed by adding or modifying internal padding. A silicone liner with a pin lock provides suspension. The iFIT prosthesis is waterproof—most conventional devices are not. The cost for an IFIT prosthesis is about one quarter the cost of a conventionally fabricated socket.

**Fig 1:**
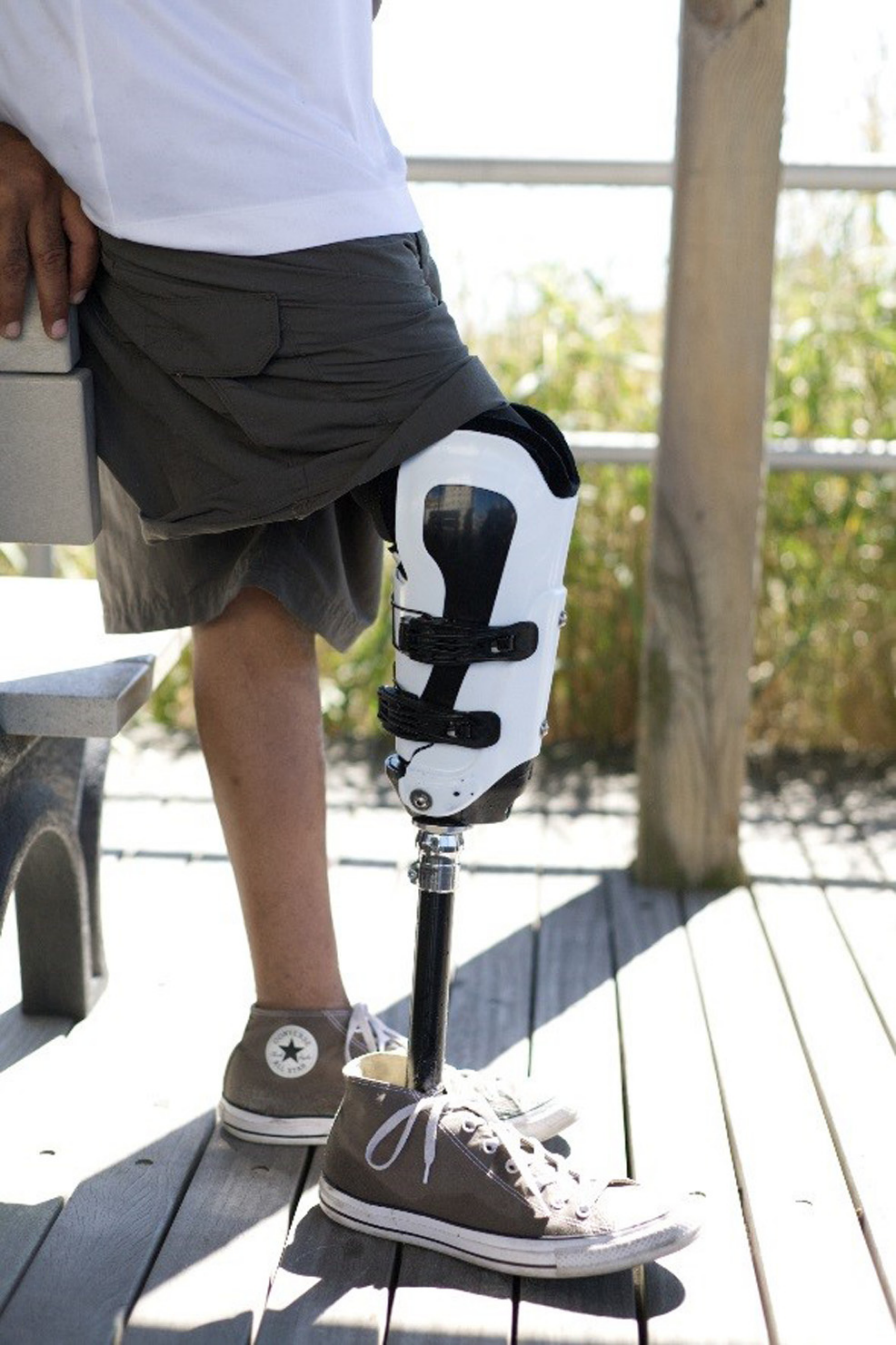
Lateral view of the iFIT prosthesis showing locking buckle closure system.

A previous study showed that the iFIT prosthesis was significantly better than patients’ conventional devices in comfort and function.^11^ The iFIT prosthesis also had lower intrasocket peak pressures than the conventional sockets and similar biomechanical gait profiles.^11^ Since this previous study, the sockets and closure systems were updated and modified to provide better internal geometry for more comfort and less bulkiness. Because of these changes, a new cohort was enlisted to evaluate the effect of these new design features on participants’ satisfaction with this modified and updated version. Our hypothesis was that the iFIT prosthesis would have better satisfaction ratings than the conventional sockets, similar to the previously conducted study.

## Methods

Volunteers with transtibial amputations were recruited through the University of Pennsylvania Health System and Philadelphia region through advertisements. Inclusion criteria were adult participants (>18y old) who had undergone transtibial amputations (both traumatic and nontraumatic); no open wounds, sutures, or staples; cleared to initiate use of a prosthesis by their surgical team or physiatrist; and intact sensation on the residual limb. Exclusion criteria included excessive phantom pain, neurologic conditions that caused marked weakness in the contralateral limb or gait abnormalities, or weight over 260 lb, which is the maximum recommended weight set by the manufacturer for commercial componentry used (pyramid connector, pylon, tube clamp) in the prosthesis. Persons who did not have a conventional prosthesis, for whom the iFIT device would be their first prosthetic device were included in this study. These individuals were included to assess this adjustable socket system when used as a preparatory prosthetic. As part of the consent process, these participants agreed to participate in outpatient physical therapy for gait training prior to using the iFIT prosthesis on their own. The therapist cleared them when they were safely ambulating. This study was approved by the University of Pennsylvania Institutional Review Board and is registered under clinical trials number NCT02886936. Participants all gave written consent prior to participation.

The primary outcome measure for this study was a modified version of the Prosthetic Evaluation Questionnaire (PEQ).^12^ This survey was modified from its original form due to length and desire to focus on socket fit and comfort rather than overall quality of life as the original survey intended. The earlier study on the initial cohort used 7 questions pulled from the PEQ which focused on comfort and stability while standing and walking with the prosthesis, making adjustments, and donning and doffing.^11^ We also asked about skin breakdown and hours per day the prosthesis was worn. The question responses were modified from the original PEQ’s visual analog scale to a 5-point rating scale with 1 equating to poor and 5 excellent to make the survey easier to complete for participants. For the present study, an additional 7 questions were added from the PEQ for this cohort (box 1). Participants filled out the survey on their conventional prosthetic during the initial visit and on the iFIT prosthetic after 2 weeks of use (participants without a conventional device only completed a survey on only the iFIT device). The participants were also asked if they had any skin breakdown, falls, how many hours per day they wore the prosthetic, and their perceptions of sweating and temperature regulation within the socket. The total score was compared, as well as each individual question to determine which questions were significant. In addition, the subset of 7 questions used for the earlier study was also calculated for the second cohort for comparison.

Participants were fit in the Physical Medicine and Rehabilitation Gait and Biomechanics Lab by the primary investigator. Participants were given a 3-mm-thick silicone locking liner (Ossur) and a foot which most closely matched their current prosthetic foot. The persons with recent limb loss who did not have a current prosthesis were given a solid ankle cushion heel foot. For the rest of the participants, they were given either a College Park Breeze foot or a College Park Celsus footb, which are both low-impact feet. The College Park Breeze foot is waterproof and given to persons that indicated they wanted to wear their prosthesis in the water. All participants were instructed on how to use the device and given a wear schedule to gradually advance wear time. Participants were scheduled to return to the Biomechanics Lab in 2 weeks to complete a second survey regarding their experience with the iFIT Prosthetic System. If a participant noticed any early alignment issues, they could return for minor adjustments to the device. Participants were allowed to keep the iFIT prostheses after the study if desired. To determine differences between the iFIT prostheses and participants’ current conventional prostheses, paired t tests were utilized. The distribution of the data was examined and found to be reasonable for parametric analysis. A nonparametric analysis resulted in similar results.

## Results

Twenty-seven participants were enrolled in the study, with 24 participants completing the 2-week study follow-up, an 89% retention rate. Three participants were lost to follow-up or did not return due to medical issues. For 3 of the 24 participants completing the study, this was their first prosthesis. These 3 participants were ambulating with a walker or crutches when they enrolled in the study—good predictors of the ability to safely use a prosthesis. They received thorough instructions on the iFIT prosthetic system and all were able to ambulate with a walker during the initial fitting session in the laboratory. At follow-up, all 3 were ambulating with assistive devices.

### Demographics

The participants were all fit during the first session and left with the iFIT transtibial prosthesis once demonstrating proficiency in its use and a stable gait. The mean age of the participants was 55.0±13.2 years old and were mostly men yet with strong female representation (22 men, 5 women [19%]). The sample was diverse with 48% African American and 11% Hispanic. The primary cause of limb loss was dysvascular disease (9 traumatic and 18 dysvascular). The majority (59.3%) used a pin suspension system, and 54.2% had carbon fiber sockets for their conventional device, and 75% reported wearing their conventional devices for 9 or more hours per day. Only 1 person had an adjustable conventional socket with some adjustability. The most frequent comorbidity reported was diabetes (66.7%). Table 1 shows the sample’s characteristics.

**Table 1:** Description of participants

**Sex**	N=27	%
Men	22	81.5
Women	5	18.5
**Ethnicity**	N=27	%
African American	13	48.1
Caucasian	11	40.7
Hispanic	3	11.1
Other	0	0
**Etiology**	N=27	%
Diabetes/vascular disease	18	66.7
Traumatic	9	33.3
**Comorbidities**	N=27	%
Diabetes	16	59.3
Heart attack	4	14.8
Cong heart failure	2	7.4
Cancer	2	7.4
Respiratory disease	2	7.4
Residual limb problem	2	7.4
Other	5	18.5
**Conventional prosthesis suspension**	n=24	%
Pin	14	58.3
Sleeve	8	33.3
Suction	2	8.3
**Type of socket**	n=24	%
Laminate	10	41.7
Carbon fiber	13	54.2
Adjustable	1	4.2
**Length of time wearing a prosthesis**	N=27	%
<1 y	8	29.6
1-10 y	14	51.9
10+	5	18.5
**Average hours/day wearing conventional**	n=24	**%**
9+	18	75
7-9	3	12.5
4-6	1	4.2
1-3	2	8.3
**Average time wearing iFIT**	n=21	%
9+	9	42.9
7-9	2	9.5
4-6	5	23.8
1-3	5	23.8

### Questionnaire results

**Fig 2:**
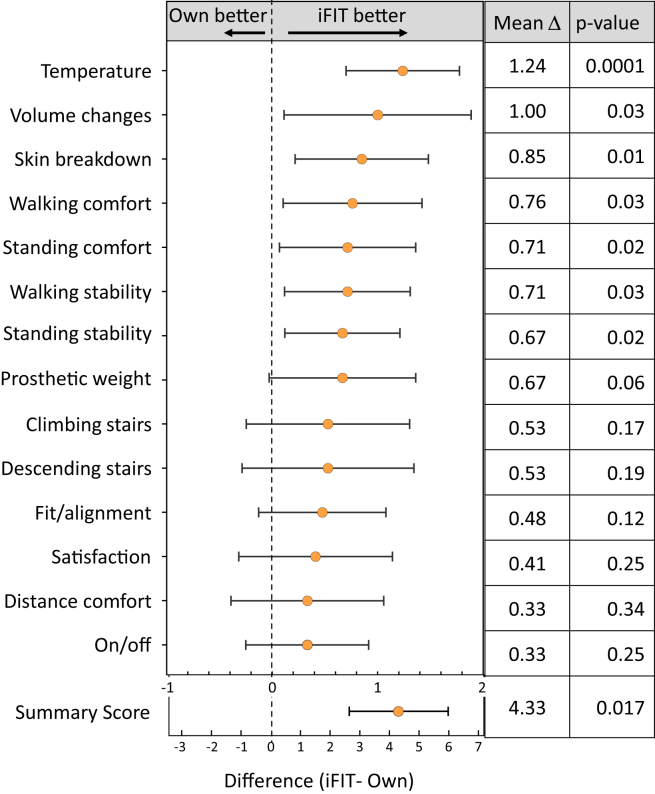
Average difference between iFIT and conventional prosthesis according to PEQ-based questionnaire data based on the 21 participants completing the study who had conventional devices prior to using the adjustable test prosthesis.

For the 21 participants who had a conventional device for comparison, we found significant differences in favor of the iFIT prosthesis in 7 of the 14 questions and on the overall modified PEQ score (fig 2). One additional category (prosthetic weight) approached significance (P=.06). Several participants omitted questions such as walking up and down stairs because they did not use stairs. However, every participant completed an answer for the 7 questions which were taken from the initial cohort.^11^ These questions were used to determine a final score used for comparison of the iFIT prosthesis to the conventional prosthetic and to compare the current study to the previous. This study found that the iFIT prosthesis was rated overall as significantly better in comparison to their conventional prosthesis (29.18±4.63 vs 23.82±6.38, P<.02). For 7 of the 14 domains, the iFIT socket was significantly better. Standing and walking comfort and stability were all rated as significantly better in favor of the iFIT socket compared to the conventional devices.

The question with greatest difference noted in favor of the iFIT socket was regarding temperature regulation where the iFIT prosthesis was rated better than their conventional sockets (4.19±0.68 vs 2.97±1.02, P<.001) in control of perceived limb temperature and sweating.

### Wear time

Daily wear time of the iFIT prosthesis and conventional devices varied. Over half the iFIT group wore the devices more than 7 hours per day (see box 1).

### Adverse events

There were no reported incidents of skin breakdown in this cohort of persons when using the iFIT prostheses. There were no mechanical issues or failures in the componentry. There were no falls reported at 2 weeks. Three people for whom the iFIT prosthesis was their first device also reported no falls or unforeseen issues. All the participants who completed the study wanted to keep their transtibial iFIT prosthesis.

### Participants who wore iFIT as their first prosthesis

The 3 participants who wore the iFIT as their first prosthetic device underwent gait training using the iFIT prostheses. As part of participation in the trial, they agreed to have physical therapy supervised gait training with the iFIT prosthesis. During the initial fitting, these participants displayed the greatest in-session volume changes, with the buckle requiring adjustments several times to accommodate the changes in the limb size decrements observed from simply walking for the first time in the laboratory. An estimated 2-3 cm of circumference was lost during initial ambulation for these participants. One of the 3 participants for whom this was the initial device completed the study and mailed in her questionnaire due to living out of state and difficulty with returning to our laboratory. This was the only participant who did not return in person for the follow-up.

These 3 participants’ daily wear time during the 2-week trial was limited because they were initially instructed to wear the prosthetic only during physical therapy and then use independently when cleared by the therapist. Their wear time was therefore not included (see box 1). During a follow-up phone call 2 months later, 2 of these participants for whom this was their first device reported they were able to wear the prosthesis the entire day. The third reported she wore the prosthesis daily for several hours.

**Box 1:** PEQ-based questions

1. Overall fit and alignment∗
2. Comfort while standing∗
3. Comfort while walking∗
4. Comfort while walking longer distances
5. Weight of the prosthesis∗
6. Stability while standing∗
7. Stability while walking∗
8. Taking the prosthesis off and putting it on∗
9. Adjusting the circumference of the device using the buckle system
10. Climbing up stairs
11. Descending down stairs
12. Temperature of the residual limb (ability of prosthesis to control excessive sweating)
13. As compared to your own device, how did the iFIT device adjust to limb fluctuations?
14. How satisfied are you overall with this prosthesis
Did you experience any skin breakdown?∗
How long did you wear the prosthesis for at the end of the trial?∗ 1-3 h 4-6 h 7-9 h 9+ h

∗ Indicates this question was asked of the initial cohort of participants.

## Discussion

In this prospective, pre-post intervention cohort study, the iFIT prosthetic system demonstrated better self-reported satisfaction scores on the modified PEQ scale in comparison to participants’ conventional prosthesis. The modified PEQ scale used in this study focuses on participants’ comfort, stability, overall fit, and ability to don/doff the prosthesis, as well as the ability to control sweating and the perception of excessive heat.

These findings are consistent with the results of a prior cohort of 22 participants who completed a similar 2-week trial with an earlier version of the iFIT prosthetic system.^11^ This study included the same group of 7 core questions based on the PEQ, yet with additional questions to assess other domains. Differences in the prosthetic ratings were remarkably similar in both studies; in the previous trial, the iFIT was rated an average of 29±4.5 points versus the conventional prosthetic 25.4±6.8, a statistically significant difference. The iFIT prosthesis in this current trial was rated as significantly better in comparison to conventional prostheses (29.18±4.63 vs 23.82±6.38, P<.02) comparable to the previous study.

The domains of walking and standing stability and comfort all were significantly better for the iFIT socket than for the conventional sockets. The iFIT sockets are designed to have supracondylar shape that firmly grasps the knee and is likely a reason for better perceived stability when standing and walking. The buckle system allows a more precise and firm closure of the socket on the residual limb. The neoprene liner in combination with the silicone suspension sleeve provides a well-padded inner socket volume to improve comfort for the participants (significantly better in the iFIT groups compared to the conventional devices).

The 10.5% rate of skin problems found in the previous study^11^ did not occur in this present study. This was likely due to the improvements made to the internal socket geometry and closure system. There was concern that the lower profile locking buckle mechanism may be difficult for participants to manipulate; however, no significant differences were found on subsection analysis for donning and doffing the prosthesis (see fig 2).

The iFIT prosthesis was rated better in terms of temperature control. Participants commented the prosthesis felt cooler with reduced sweating and heat buildup in their residual limbs. Increased perspiration using a silicone liner is common in prosthetics,^13^ and improved sweat management can increase prosthetic satisfaction.^14^ The perceived improvement in temperature regulation with the iFIT socket is likely related to several design features. The perforated inner neoprene liner allows some airflow between the silicone suspension sleeve and the adjustable socket. Participants using the iFIT prosthesis use a thinner silicone suspension sleeve (3mm) which can more easily dissipate heat. Many conventional sockets require patients to use thick (6-9mm) silicone liners to achieve a comfortable fit. Sleeve suspension systems in conventional devices trap heat in the socket and were used by 33% of our sample for their conventional device. Suction suspension systems have rings around the sleeve to provide a firm vacuum, also trapping heat. In this cohort, 41.6% of patients had sleeve or suction suspension systems. In the iFIT socket, the rear overlapping flaps have a space at the bottom for air to flow around the residual limb, offering additional cooling and ventilation.

Although iFIT prostheses received better ratings, decreased wear time was reported for the iFIT prosthetic in comparison to the conventional prostheses. This was possibly due to difficulty getting tight clothing over the socket in some cases. Patients have reported that it is somewhat bulkier than their conventional devices. In addition, participants frequently commented on the differences (eg, *stiffness*) of our study prosthetic feet relative to the prosthetic feet on their conventional devices. Many participants wore different feet than what was provided in this study, for example, dynamic response and higher performance feet. The study feet were selected for performance and cost characteristics.

The iFIT system is intended to provide an affordable and accessible alternative to conventionally fabricated prosthetic systems. The iFIT device provides an alternative to conventional devices because it is lower in cost, fits in 1 session, and provides better comfort and patient satisfaction.

The iFIT transtibial prosthetic can be fit and aligned in a single session, allowing patients to begin using a prosthetic system as soon as they are cleared by their physician, facilitating early gait training and rehabilitation. A recent Veteran’s Administration Guideline for care highlighted the beneficial effects of early ambulation to improve functional status and enhance psychological well-being.^15^ In addition, the lower cost and ability to fit without a full prosthetic lab make the iFIT prosthetic an option for the large population of persons with lower limb loss in the developing world that currently are functioning without lower limb prostheses due to access and cost issues.^8^

Three participants in this trial used the iFIT prosthesis as their first prosthetic device. These participants readjusted the socket multiple times during the first use in our lab because the limb volume changed considerably with initial ambulation. The conventional method for managing volume loss is to add layers of socks in order to obtain a proper fit. Most of the participants in this trial had limb loss resulting from dysvascular causes (diabetes and peripheral artery disease). This is similar to previous research studies that found 54%-82% of persons with limb loss are due to vascular disease.^1^^,^^16^ The ability to adjust the prosthesis day to day is beneficial for patients who experience frequent volume changes, such as those in their first year postamputation or those patients with heart or renal diseases that cause fluctuations in volume status.

This cohort consisted of a diverse sample with sex and racial representation (see box 1). We chose a follow-up duration of 2 weeks to ensure a high rate of study retention for this cohort of patients. This study duration is long enough to allow patients to assess comfort and functionality of the prosthesis and enhance study completion.

These devices have been used in a cohort of persons with transtibial limb loss in Jamaica and demonstrated long-term durability (>2y) as well as high patient satisfaction (J. Kenia et al, unpublished data, 2020). iFIT sockets underwent cyclic testing using the International Organization for Standardization (10328—structural testing of lower limb prostheses) standards for repetitive stresses (conditions I and II—300 lb for 3 million cycles) without breakage.^17^ The socket also exceeded maximum recommended component failure stresses as specified by International Standardization Organization testing guidelines.^17^

### Study limitations

This study is not without some limitations. The participants in this study were all volunteers who were interested in trying an alternative prosthesis. They may have experienced some degree of dissatisfaction with their conventional prosthesis. In addition, there was a small group of participants who were unable to get a prosthesis at all without the study due to insurance denials and lack of resources. We also were unable to fully assess and match the foot worn by the participant while using their conventional socket. Last, the study featured a small population, fit through a single center, with limited follow-up time. Long-term follow up with a larger population is needed to more fully assess the results seen in this study.

## Conclusions

The iFIT immediate fit, adjustable transtibial prosthesis demonstrated significantly better self-reported comfort and patient satisfaction in this prospective study than conventionally fabricated prostheses. The results of this study are consistent with those from a previous investigation and add to a growing body of work demonstrating the safety and feasibility of this adjustable transtibial prosthetic system.^11^ This system can benefit persons with limb loss during the first year after amputation when the limb rapidly changes in volume. It can also serve as a definitive device for individuals with heart and/or renal disease who experience daily limb volume fluctuations. Children and teens with residual limbs that are expected to grow may also benefit from an adjustable socket. Persons publicly insured or without insurance can get the iFIT device for less out-of-pocket expenses and expect a high level of comfort and functionality.

## Suppliers

a. iFIT transtibial prosthesis; iFIT Prosthetics, LLC.
b. College Park Breeze, College Park Celsus; College Park Industries.
